# Hot tensile properties and constant load stress corrosion cracking test data of autogenous weld joints of super 304HCu stainless steel in boiling MgCl_2_ solution

**DOI:** 10.1016/j.dib.2018.03.002

**Published:** 2018-03-07

**Authors:** M. Vinoth Kumar, V. Balasubramanian

**Affiliations:** a Department of Mechanical Engineering, Hindustan Institute of Technology and Science, Padur, Chennai, Tamilnadu 603103, India; b Department of Manufacturing Engineering, Annamalai University, Annamalai Nagar, Tamilnadu 608002, India

## Abstract

Hot tensile test data of Gas Tungsten Arc Welded (GTAW) autogenous joints of Super 304HCu tubes tested at their operating temperature are presented along with the microstructure of the weld joint. The GTAW joints exhibited lower tensile strength than the parent metal and the failure occurred in the weld metal region for all test temperatures. Constant load Stress Corrosion Cracking (SCC) test data of the GTAW weld joints tested in boiling MgCl_2_ environment at different applied stress level are presented. SCC curves obtained from the test were analyzed to derive SCC parameters such as rate of steady state elongation, the time required for set-in of tertiary region, and time to complete fracture. The fracture surfaces of SCC samples were examined using Scanning Electron Microscope to reveal the mode of fracture. Super 304HCu stainless steel being used as construction material for super heaters and reheaters of advanced ultra super critical boilers, this data will be an addition to the design data available for material selection in design of power plants.

**Specifications table**

**Table:** 

Subject area	*Metallurgy, Corrosion Science*
More specific subject area	*Welding, Destructive Testing, Stress Corrosion Cracking*
Type of data	*Table, microscopy, graph, figure*
How data was acquired	*Optical Microscope, Scanning Electron Microscope, Energy Dispersive Spectroscopy, Universal Testing Machine, Stress Corrosion Cracking Test*
Data format	*Filtered and Analyzed*
Experimental factors	*Super 304HCu tubes autogenously welded and specimens for testing are extracted by machining*
Experimental features	*High Temperature Tensile Test as per ASTM E21, Stress Corrosion Cracking Test as per ASTM G36*
Data source location	*Annamalai University, Chidambaram, Tamil Nadu, India, 608002.*
Data accessibility	*None*

**Value of the data**

• *Microstructural variation in the autogenous GTA weld joint of super 304HCu with details of micro segregation in the weld metal*
• *High temperature tensile test data of cross weld samples of autogenous GTA welded joints of super 304HCu can be used in accessing the structural integrity of the welded joint*
• *SCC rate of steady state elongation can be used to predict the remaining time to failure of the components in service*
• *Provided data can be used to determine the cracking mechanisms associated with the SCC failure*

## 1 Data

Super 304HCu (Cr-Ni-Cu-N-Nb-B) austenitic stainless steel tubes with distinct addition of 2.3 to 3 (% wt) of copper is a candidate material for use in super heaters and reheaters of advanced ultra super critical nuclear power plants [1]. A high degree of chemical compatibility between the construction materials and the operating fluids / environment, to avoid stress corrosion cracking (SCC) needs to be ensured [2]. Stainless steels in chloride environment are susceptible to SCC, and evaluation of the SCC behaviour of the fabricated weld joints becomes inevitable. Welding is considered as the major manufacturing methods for pressure equipment's in power plants [3]. Welding may alter the favorable parent metal microstructure and induce residual stresses in the joints [4]. Fusion welding alters the phase composition, and microstructure of the steel and such alteration can affect the mechanical and corrosion characteristics in contrast to the parent material. Chloride stress corrosion cracking (SCC) is the most likely life limiting failure in austenitic stainless steel tubing of USC boilers and welding can even worsen the SCC susceptibility of the material [5]. The SCC experiments are conducted on autogenous GTA welded Super 304HCu joint in 45% boiling MgCl_2_ solution using constant load SCC and their respective corrosion elongation curves are presented. The structural integrity of the autogenous joint evaluated at their operating temperatures using high temperature tensile tests are presented.

## 2 Experimental design, materials and methods

Super 304H tubes of 57.1 mm diameter and 3.5 mm thick were used as base material in this investigation. The chemical composition of the tubes in as-received condition is given in Table 1.

**Table 1:** Chemical composition (wt %) of Super 304HCu.

C	Si	Mn	P	S	Cr	Ni	N	Cu	Nb	B	Al
0.086	0.23	0.81	0.021	0.0003	18.18	9.06	0.095	3.080	0.045	0.0039	0.01

The joints with square butt edge preparation were welded using PC-GTAW processes with argon as the shielding and purging gas. The welding parameters used in this investigation are shown in Table 2.

**Table 2:** GTAW welding parameters.

Current mode	Pulsed
Peak current (A)	110
Base current (A)	66
% on time	75
Frequency (Hz)	10
Voltage (V)	11
Welding speed (mm/min)	70
Heat input (kJ/mm)	0.933

The weld joints were inspected for full penetration and the test samples are extracted using wire-cut electric discharge machining.

### 2.1 Tensile properties

**Fig. 1:**
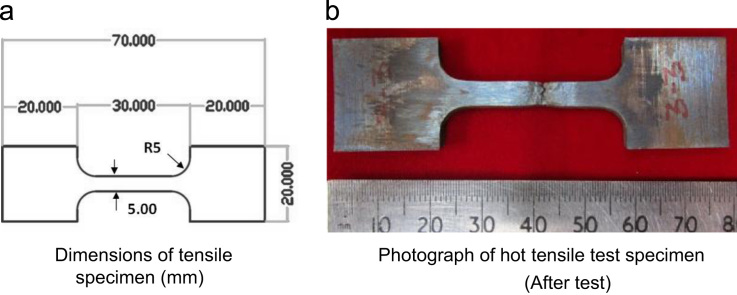
Details of the tensile specimen.

The dimensions of tensile specimen along with the photograph of the specimen after test is shown in Fig. 1. Tensile tests were carried out using Instron make universal testing machine (UTM), at four different temperatures, such as room temperature (RT), 550 °C, 600 °C, and 650 °C under a nominal strain rate of 10^−3^ s^−1^ as per ASTM E21 standard [6]. The UTM was equipped with a three-zone resistance heating furnace for high temperature tests and a computer with data acquisition system for obtaining digital load-elongation data.

**Fig. 2:**
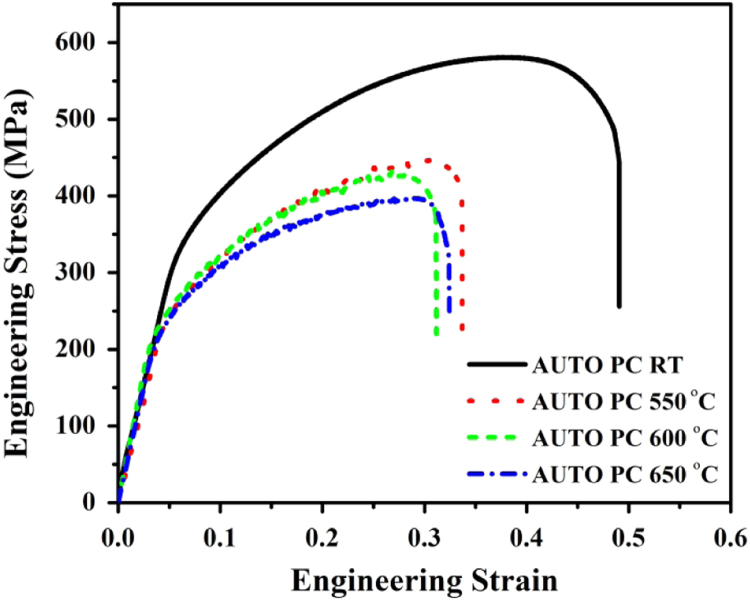
Engineering stress-strain curves of autogenous GTAW weld joint of Super 304H at different test temperature.

The engineering stress-strain curves of autogenous GTAW joints of Super 304HCu at various test temperatures are shown in Fig. 2 and their tensile properties are presented in Table 3. The tensile strength of weld joint decreases with increase in test temperature and failure is observed in the weld metal region at all test temperature.

**Table 3:** Tensile properties of parent metal and autogenous GTAW joints of Super 304HCu.

Material	Test temperature (°C)	Yield strength (MPa)	Ultimate tensile strength (MPa)	Elongation (%)	Failure location
Parent metal [7]	RT	284.2	575.8	71.8	–
Weld joint	RT	–	570	39.2	Weld metal
	550	–	444	38.6	Weld metal
	600	–	425	35.1	Weld metal
	650	–	395	39.4	Weld metal

### 2.2 Microstructure

The metallographic samples were prepared using standard metallographic techniques and etched with Glyceregia (15 ml glycerol, 10 ml HCl, and 5 ml HNO_3_) for 5–10 s to reveal the general structure of parent metal and with boiling Murakami's reagent (10 g KOH, 10 g potassium ferricyanide, 100 mL water) to reveal δ ferrite and carbides in the weld metal. The microstructural examination of the samples were carried out using light optical microscope (OM), scanning electron microscope (SEM) and compositional variation within the weld regions are determined using energy dispersive spectroscopy (EDS) attached with SEM.

**Fig. 3:**
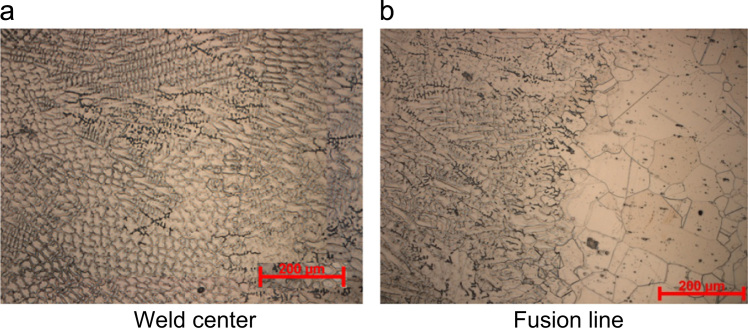
Optical micrographs of autogenous GTAW joints of Super 304HCu.

Optical micrographs of weld center of the GTAW joint is shown in Fig. 3a, which reveals austenite grains of both cellular and columnar morphology. The intercellular and interdendritic region reveals a dark phase preferably eutectic delta ferrite. The fusion line micrograph (Fig. 3b) reveals the grain coarsening in the HAZ.

**Fig. 4:**
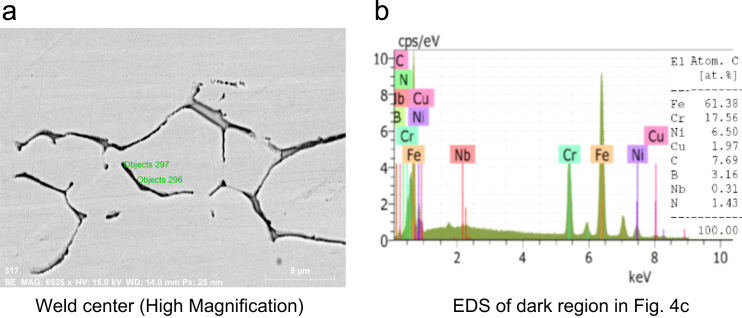
SEM micrograph and EDS analysis of autogenous GTAW joint of Super 304HCu.

SEM micrographs of the weld center etched with murakami's reagent is shown in Fig. 4a, reveals 2 distinct phases, an austenite (dark phase) and a segregated phase (bright phase). The EDS spectrum of intercellular dark phase is shown in Fig. 4b reveals increased levels of B, C and reduced level of austenite forming elements in the segregated phase than the matrix, which allows to infers that the interdendritic regions consists of delta ferrite with borocarbides precipitates.

### 2.3 Stress corrosion cracking

**Fig. 5:**
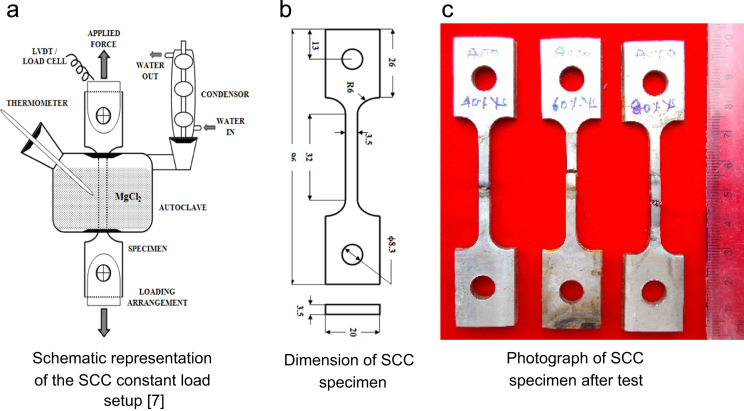
Details of SCC test setup and specimen.

SCC tests were carried out in a custom built constant load SCC setup and the schematic representation of the setup is shown in Fig. 5a. The dimensions of the smooth tensile SCC specimen is shown in Fig. 5b. The maximum loading capacity of the setup was 10 kN and the applied load was measured using a load cell with an accuracy of ± 10 N. The strain measurements were recorded using a LVDT with measurable range of ± 5 mm and an accuracy of < 1 µm. The smooth tensile specimens after constant load SCC test are shown in Fig. 5c.

**Fig. 6:**
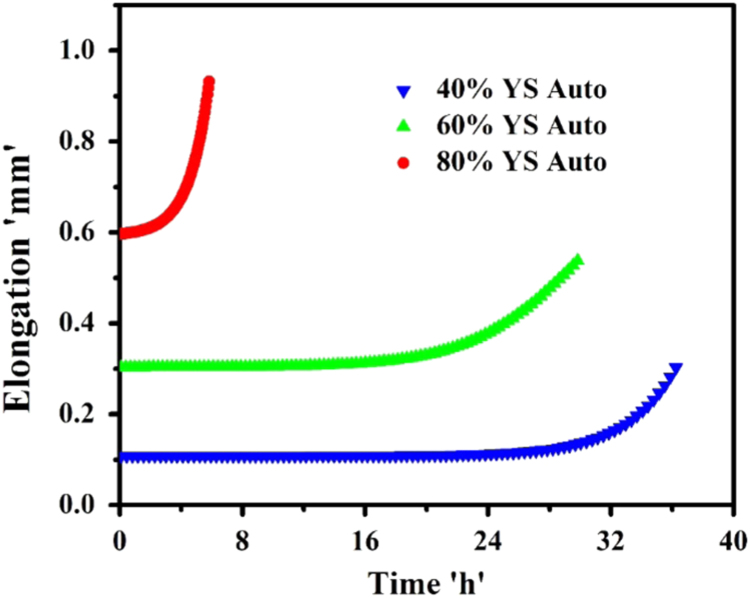
Corrosion elongation curves for autogenous GTAW joint of Super 304HCu at different levels of applied stress.

The environment for SCC testing of Super 304H was chosen as 45% MgCl_2_ boiling at 155 °C, and the tests were conducted in accordance with ASTM G36 [8]. The constant load SCC tests were conducted at applied stress levels of 1.0, 0.8, 0.6, and 0.4 times of parent metal yield strength. The corrosion elongation curves at different applied stress levels are shown in Fig. 6 and their parameters are presented in Table 4.

**Table 4:** SCC corrosion elongation curve parameters of autogenous GTAW joints of Super 304HCu.

Specimen	% of applied tensile stress	Time to failure (t_f_) ‘h’	Time to transition (t_ss_) ‘h’	t_ss_ / t_f_	Elongation rate (i_ss_) ‘mm/s’
Weld joint	0.8 × YS	5.83	–	–	–
	0.6 × YS	29.83	17.51	0.587	2.13E-09
	0.4 × YS	36.25	27.48	0.758	1.15E-10

From corrosion elongation curve, parameters such as i_ss_, t_ss_ and t_f_ can derived where, i_ss_ is the slope of the curve in secondary region before the time to transition (t_ss_) from secondary to tertiary region, representing the rate of steady state elongation, t_ss_ is the time required for set-in of tertiary region, t_f_ is the time to complete fracture. The ratio of t_ss_ to t_f_ is used to determine the most prominent mode of degradation (Corrosive or Mechanical) active in their respective test conditions.

The value of t_ss_/t_f_ tends to increase with decrease in applied stress which implies that the time in tertiary region i.e. the time to fracture after crack initiation decreases steadily with decrease in applied stress level, whereas the steady state elongation rate (i_ss_) decreases with increase in t_f_ and decrease in applied stress. SCC is rapid at 0.8×YS of applied stress indicated by its corrosion elongation curve which does not reveal region of steady state elongation. Hence, the SCC test is not carried out at applied stress of 1.0×YS.

**Fig. 7:**
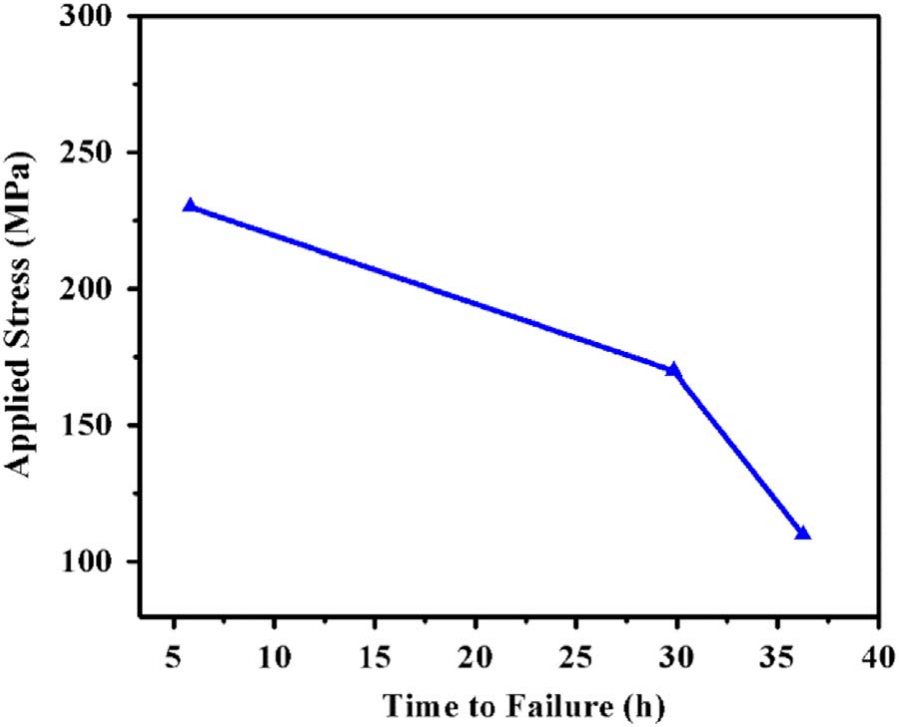
Relationship between applied stress and time to failure by SCC for autogenous GTAW joints of Super 304HCu.

The relationship between applied stress and time to failure (t_f_) is shown in Fig. 7. It can be inferred that a break down in the relationship was observed at 0.6×YS of the applied stress. The breakdown suggests the change in SCC mechanism which is more prominently active. The applied stress level of 0.4×YS and 0.6×YS falls in the range where the SCC is dominated by corrosion, at applied stress level greater than 0.6×YS the SCC mechanism is dominated by stress.

### 2.4 Fracture surface

**Fig. 8:**
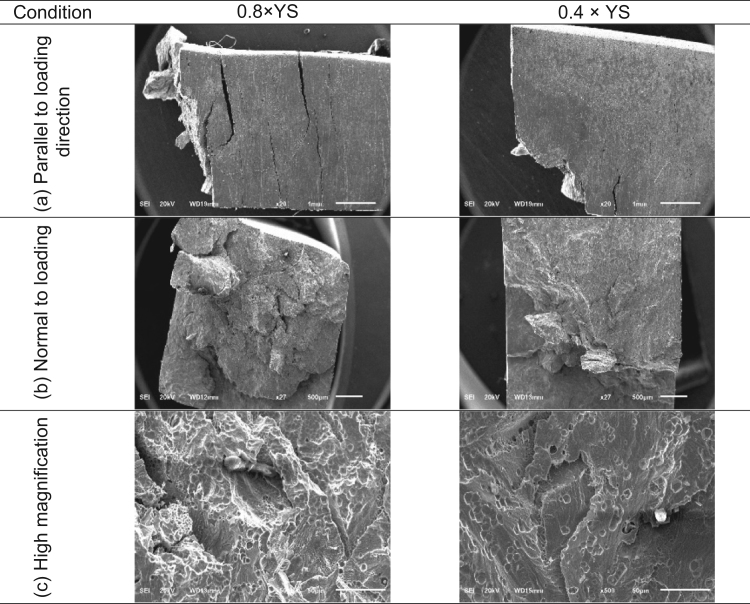
Fracture surface of SCC specimen.

Fracture surface of SCC specimens tested at different applied stress level are shown in Fig. 8. The fractured portion of specimen along the loading direction, shown in Fig. 8a reveals the relationship between number of crack and the applied stress level. Number of cracks increases with increase in applied stress level and the direction of crack propagation is normal to the direction of applied stress. Fig. 8b reveals the completely brittle nature of the SCC in Super 304HCu joints and the high magnification image shown in Fig. 8c reveals transgranular mode of crack propagation and decohesion in the grain boundaries.

**Fig. 9:**
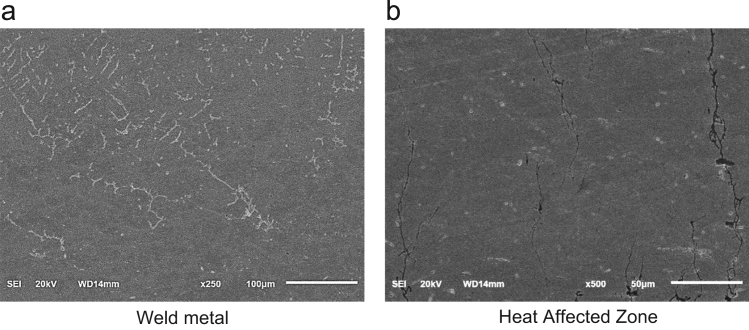
SCC characteristics of crack propagation in autogenous GTAW weld joint of Super 304HCu tested in corrosion dominant region (40% YS).

SCC behaviour of autogenous GTAW joint in corrosion dominant region is studied using SEM micrographs. SEM images of weld metal and HAZ of specimen tested at (0.4×YS) in corrosion dominant region are shown in Fig. 9. The weld metal and fusion line of the joint shown in Fig. 9a reveals no cracking or corrosion sites in the weld metal region. The HAZ next to the fusion line (refer Fig. 9b) reveals cracking, with cracks initiating from the surface of the specimen and running across the thickness of the specimen, with lesser branching, attributed to lower level of applied stress.
